# Angiography-Derived Microcirculatory Resistance in Detecting Microvascular Obstruction and Predicting Heart Failure After STEMI

**DOI:** 10.1161/CIRCIMAGING.124.017506

**Published:** 2025-04-03

**Authors:** Guanyu Lu, Lei Zhao, Keyao Hui, Zhihui Lu, Xiaoli Zhang, Hai Gao, Xiaohai Ma

**Affiliations:** 1 Department of Interventional Diagnosis and Treatment (G.L., K.H., Z.L., X.M.), Beijing Anzhen Hospital, Capital Medical University, China.; 2 Department of Radiology (L.Z.), Beijing Anzhen Hospital, Capital Medical University, China.; 3 Department of Cardiology, Emergency Coronary Artery Unit (H.G.), Beijing Anzhen Hospital, Capital Medical University, China.; 4 Department of Nuclear Medicine, Molecular Imaging Laboratory (X.Z.), Beijing Anzhen Hospital, Capital Medical University, China.

**Keywords:** angiography, heart failure, magnetic resonance imaging, myocardial reperfusion, prognosis

## Abstract

**BACKGROUND:**

Microvascular obstruction (MVO) is associated with heart failure (HF) following ST-segment–elevation myocardial infarction. Angiography-derived microcirculatory resistance (AMR), a wire- and adenosine-free measure, may facilitate early assessment of microvascular function post-primary percutaneous coronary intervention. This study aimed to evaluate the ability of AMR to detect MVO and its prognostic value for predicting HF in patients with ST-segment–elevation myocardial infarction post-primary percutaneous coronary intervention.

**METHODS:**

Patients with consecutive ST-segment–elevation myocardial infarction undergoing primary percutaneous coronary intervention with a cardiac magnetic resonance examination 2 to 7 days post-procedure between April 2016 and February 2023 were retrospectively reviewed. AMR was computed from coronary angiography. MVO was identified and quantified via cardiac magnetic resonance. The end point was new-onset HF during follow-up.

**RESULTS:**

Overall, 475 patients (aged 56.8±11.7 years; 399 men) were included. The area under the curve for AMR to detect MVO was 0.821 (95% CI, 0.782–0.859), with an optimal cutoff value of 2.7 mm Hg*s/cm. During a median follow-up of 37.3 months, 121 (25.5%) patients developed HF. AMR, whether as a continuous (per 0.5-mm Hg*s/cm increase; hazard ratio, 1.29 [95% CI, 1.10–1.52]; *P*=0.002) or categorical (AMR >2.7 mm Hg*s/cm; hazard ratio, 2.15 [95% CI, 1.43–3.22]; *P*<0.001) variable, was independently associated with HF after adjusting for traditional risk factors (age, symptom-to-balloon time, left anterior descending coronary artery, and ejection fraction) and late gadolinium enhancement-cardiac magnetic resonance parameters. AMR improved prognostication over traditional risk factors and late gadolinium enhancement-cardiac magnetic resonance parameters (net reclassification improvement, 0.533; *P*<0.001; integrative discrimination index, 0.023; *P*=0.005).

**CONCLUSIONS:**

AMR showed good diagnostic performance in detecting MVO and was an independent and incremental predictor of HF in patients with ST-segment–elevation myocardial infarction post-primary percutaneous coronary intervention.

**Clinical Perspective**

Angiography-derived microcirculatory resistance (AMR), a wire- and adenosine-free measure, offers a novel approach for the early assessment of microvascular function following primary percutaneous coronary intervention. In this study, we evaluated the diagnostic and prognostic utility of AMR in patients with ST-segment–elevation myocardial infarction after successful primary percutaneous coronary intervention. Our findings demonstrated that AMR exhibited good diagnostic performance in detecting microvascular obstruction, with an optimal cutoff value of 2.7 mm Hg*s/cm. Moreover, AMR was a powerful independent predictor of heart failure. For every 0.5-mm Hg*s/cm increase in AMR, the risk of heart failure increased by 29%. Patients with elevated AMR (>2.7 mm Hg*s/cm) had a 2.15-fold higher risk of heart failure compared with those with low AMR. Furthermore, AMR provided incremental prognostic information beyond traditional risk factors (age, symptom-to-balloon time, left anterior descending coronary artery as the infarct-related artery, and left ventricular ejection fraction) and late gadolinium enhancement-cardiac magnetic resonance parameters. Given its simplicity, quantitative nature, and feasibility, AMR represents a promising tool for early risk stratification in ST-segment–elevation myocardial infarction management, potentially facilitating a triage of high-risk patients toward more aggressive therapeutic interventions and intensive follow-up.

Despite advancements in prompt coronary reperfusion via primary percutaneous coronary intervention (PPCI), heart failure (HF) incidence following ST-segment–elevation myocardial infarction (STEMI) has not declined and may be increasing.^1,2^ HF after STEMI is associated with a higher risk of mortality, highlighting the importance of risk prediction for HF.^3,4^ Approximately half of patients with STEMI exhibit suboptimal myocardial reperfusion, even after timely PPCI to restore epicardial blood flow.^5,6^ Microvascular obstruction (MVO) plays a deleterious role in the resulting suboptimal reperfusion and myocardial structural damage and is associated with the occurrence of HF.^5,6^

Cardiac magnetic resonance (CMR) imaging is considered the gold standard for assessing MVO.^7,8^ However, routine CMR, typically performed days after STEMI, cannot guide additional therapies during the PPCI procedure to prevent persistent microvascular damage.^9^ In addition, it is not always available and cost-effective.^9^ The wire-based index of microcirculatory resistance (IMR) facilitates the assessment of microvascular dysfunction immediately after stenting in STEMI.^10^ This measure has been proven to characterize MVO and is associated with subsequent HF and cardiac death.^11–13^ Nonetheless, the clinical application of IMR remains limited, primarily due to the requirement of both pressure-temperature sensor wires and hyperemic agents, which increases the complexity and duration of the PPCI procedure.^9^

Recent advancements in angiography-derived physiology have enabled the estimation of IMR using angiographic data alone.^9^ Among these methods, angiography-derived microcirculatory resistance (AMR), a wire- and adenosine-free index, is computed based on Murray law–based quantitative flow ratio from a single angiographic view.^14,15^ AMR has shown promising accuracy in traditional wire-based IMR computational estimates.^15^ Moreover, it overcomes the limitations of the 2-view angiography-derived IMR, which often fails to analyze nearly half of cases due to the absence of a second appropriate angiographic view.^16,17^ However, research on AMR is in its early stages, and the ability of AMR to detect MVO and its prognostic value in predicting subsequent HF for patients with STEMI undergoing PPCI remains unclear.

This study aimed to investigate the feasibility of AMR to assess MVO and its prognostic significance in predicting HF in patients with STEMI following PPCI.

## Methods

Data supporting the findings of this study are available from the corresponding author upon reasonable request. This study was approved by the institute review board of our hospital (2024125X). The requirement for written informed consent was waived owing to the study’s retrospective nature.

### Study Population

Data of consecutive patients admitted for a first STEMI and undergoing PPCI, followed by a CMR examination 2 to 7 days post-procedure, between April 2016 and February 2023 at a tertiary cardiac center, were retrospectively reviewed. STEMI was diagnosed according to the fourth universal definition of myocardial infarction.^18^ PPCI was performed using standard procedures. Stenting type, thrombectomy, and glycoprotein IIb/IIIa inhibitor use were at the operator’s discretion. Exclusion criteria were incomplete clinical records (n=5); left main coronary artery lesions (n=3) and thrombolysis in myocardial infarction flow grade <3 post-procedure (n=9) for reliable AMR measurement; HF history (n=7); gadolinium-based contrast agent not administered (n=5); poor image quality for functional angiographic analysis (n=2) and CMR analysis (n=4); and loss to follow-up (n=8; Figure S1).

### AMR Measurement

AMR measurement was performed in the infarct-related artery on the final angiogram after successful revascularization. Angiograms were obtained after nitroglycerin administration in all cases. A single-view AMR analysis was conducted as previously described,^14,15^ using AngioPlus Core software (version V3; Shanghai Pulse Medical Technology, Inc, Shanghai, China) by 2 experienced and certified analysts blinded to CMR and clinical data. AMR measurement details are described in the Supplemental Methods.

### CMR Acquisition Protocol and Analysis

CMR was performed using a 3.0-T scanner (Magnetom Verio, Siemens Health Care, Erlangen, Germany; Ingenia, Philips Healthcare, Best, the Netherlands; or Discovery MR750, General Electric Healthcare, Milwaukee, WI). Cine images were acquired using a balanced steady-state free precession sequence in the short axis, covering the entire ventricle from the apex to the atrioventricular valve plane and in 3 long-axis views (2, 3, and 4 chambers). Late gadolinium enhancement (LGE) imaging was performed 10 to 15 minutes after administering a 0.2-mmol/kg gadolinium-based contrast agent (gadopentetate dimeglumine, Magnevist, Bayer Schering, Germany) using a phase-sensitive inversion-recovery sequence in views identical to cine CMR imaging. Detailed CMR acquisition parameters are listed in Table S1.

CMR analyses were conducted using CVI42 software (version 5.11.2; Circle Cardiovascular Imaging, Inc, Calgary, AB, Canada) by 3 experienced radiologists (>5 years of experience in CMR diagnosis) blinded to AMR and clinical data. Infarct size (% of left ventricular [LV] mass) and MVO extent (% of LV mass) were quantified on LGE imaging. Detailed CMR analyses are described in the Supplemental Methods.

### Study Outcomes

Follow-up data were collected through medical records, clinic evaluations, and telephone interviews. Patients who missed regular hospital visits were contacted via phone, and their medical records were requested from other hospitals to determine whether they had reached end points. The study end point was the first occurrence of new-onset HF following PPCI. New-onset HF was defined as the need for increased HF therapy in the appropriate clinical setting or HF hospitalization.^4^ Detailed definition is provided in the Supplemental Methods. Cardiac mortality was recorded. All adjudications of events were conducted by 2 experienced cardiologists (with 20 and 18 years of experience in diagnosing and treating cardiovascular diseases, respectively) blinded to AMR and initial CMR results.

### Statistical Analysis

Normality was verified using the Shapiro-Wilk test. Continuous variables are expressed as mean±SD or median (interquartile range [IQR]). Categorical variables are expressed as numbers and percentages. Comparison between 2 groups was performed using the Student *t* test, the Mann-Whitney *U* test, the Fisher exact test, or the χ^2^ test, accordingly. Correlation between variables was tested with the Pearson r coefficient. The area under the receiver operating characteristic curve was used to evaluate the diagnostic performance of AMR to detect MVO on CMR. The optimal cutoff value of AMR for detecting MVO maximized sensitivity and specificity, as determined by receiver operating characteristic curve analysis. For the reproducibility analysis of AMR, angiograms from 30 patients randomly selected from the cohort were analyzed by the same blinded analyst 2 weeks later to assess intraobserver agreement and independently by a second blinded analyst to assess interobserver agreement. Intraobserver and interobserver agreements were assessed using the Bland-Altman analysis. Survival curves were obtained by the Kaplan-Meier analyses and compared using log-rank tests. Follow-up time was calculated from the date of PPCI to the date of the first new-onset HF, censoring patients who were alive without HF at the latest follow-up date. Cox proportional hazards regression analyses were conducted to investigate associations with HF. To evaluate the prognostic value of AMR, we established 6 different multivariable models that included variables with *P*<0.05 on univariable screening as covariates. Given that AMR and LGE-CMR parameters could characterize MVO, we excluded them from the baseline multivariable model (model 1). Subsequently, AMR, LGE-CMR parameters, and their combination were added to model 1 to form additional models. The specific models are given as follows: model 1 (baseline), traditional risk factors with *P*<0.05 on univariable screening, including age, symptom-to-balloon time, left anterior descending coronary artery as the infarct-related artery, and LV ejection fraction; model 2, model 1 plus AMR as a continuous variable; model 3, model 1 plus AMR as a categorical variable (AMR >2.7 mm Hg*s/cm); model 4: model 1 plus LGE-CMR parameters (infarct size and MVO extent); model 5, model 1 plus LGE-CMR parameters and AMR as a continuous variable; and model 6, model 1 plus LGE-CMR parameters and AMR as a categorical variable. Model performances were evaluated and compared using the Harrell C statistic, continuous net reclassification improvement (NRI), and integrated discrimination improvement (IDI). No evidence of problematic strong collinearity was found among the covariates in the multivariable models. The proportional hazards assumption was met. *P*<0.05 was considered significant. Statistical analyses were performed using SPSS, version 26.0 software (IBM, Armonk, NY); GraphPad Prism, version 7.0 (GraphPad Software, San Diego, CA); R, version 4.3.3 (R Project for Statistical Computing); and MedCalc statistical software.

## Results

### Overall Characteristics of the Study Population

Of the 518 patients admitted for a first STEMI who underwent PPCI and a CMR examination 2 to 7 days post-procedure, 43 patients were excluded based on exclusion criteria. Overall, 475 patients with STEMI were ultimately included (Figure S1). Tables 1 and 2 show the characteristics of the study population. The mean age was 56.8±11.7 years, and 399 of 475 (84.0%) patients were men.

**Table 1.**
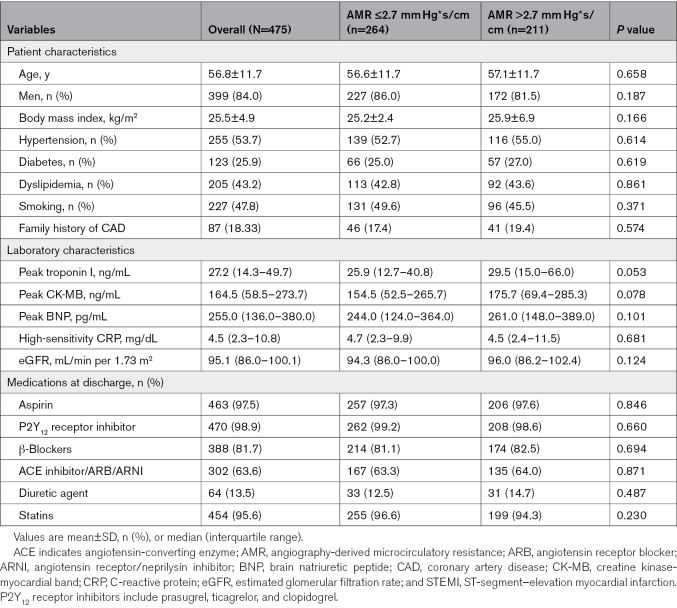
Clinical Characteristics of 475 Patients With STEMI According to AMR

**Table 2.**
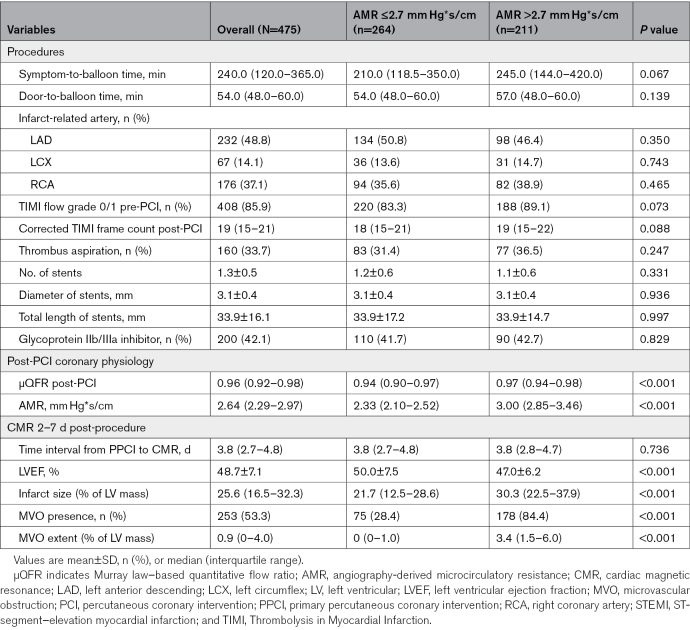
Procedural, Physiology, and CMR Data of 475 Patients With STEMI According to AMR

### Diagnostic Performance of AMR to Assess MVO

The mean time for measuring single vessel AMR was 2.26±0.90 minutes. The median AMR was 2.64 (IQR, 2.29–2.97) mm Hg*s/cm. The median time from PPCI to CMR was 3.8 (IQR, 2.7–4.8) days. Infarct size and MVO extent were 25.6% (IQR, 16.5%–32.3%) and 0.9% (IQR, 0%–4.0%), respectively.

**Figure 1.**
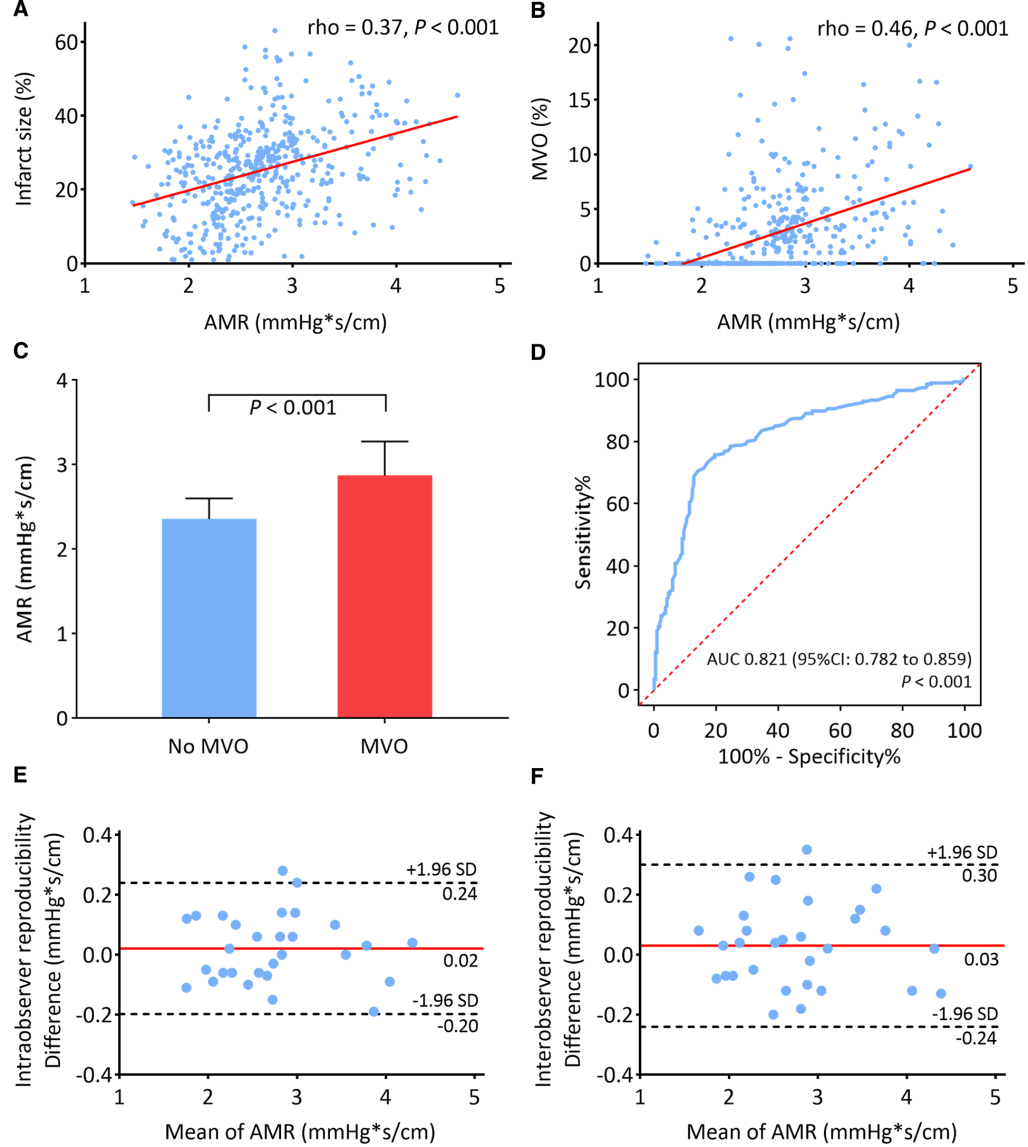
**Angiography-derived microcirculatory resistance (AMR) diagnostic performance to assess microvascular obstruction (MVO) in patients with ST-segment–elevation myocardial infarction (STEMI) post-primary percutaneous coronary intervention. A**, Scatterplot showing the correlation between AMR and infarct size. **B**, Scatterplot showing the correlation between AMR and MVO. **C**, Bar chart displaying median AMR values in patients with and without MVO on cardiovascular magnetic resonance. **D**, Receiver operating characteristic curve analysis of AMR for detecting MVO. **E**, Bland-Altman plots for intraobserver reproducibility of AMR. **F**, Bland-Altman plots for interobserver reproducibility of AMR. AUC indicates area under the curve.

AMR showed a significant correlation with infarct size (ρ=0.37; *P*<0.001) and MVO extent (ρ=0.46; *P*<0.001; Figure 1A and 1B). Patients with evidence of MVO exhibited a significantly higher AMR value than that of those without (2.87 [IQR, 2.65–3.28] versus 2.36 [IQR, 2.14–2.60] mm Hg*s/cm; *P*<0.001; Figure 1C). In the receiver operating characteristic curve analysis, the area under the curve of AMR for detecting MVO was 0.821 (95% CI, 0.782–0.859), with an optimal cutoff value of 2.7 mm Hg*s/cm (Figure 1D). The sensitivity, specificity, positive predictive values, negative predictive values, and diagnostic accuracy of AMR >2.7 mm Hg*s/cm for detecting MVO were 70.4%, 85.1%, 84.4%, 71.6%, and 77.3%, respectively.

Overall, 211 (44.4%) patients exhibited high AMR (>2.7 mm Hg*s/cm), and 264 (55.6%) showed low AMR (≤2.7 mm Hg*s/cm) at the completion of PPCI. Based on the AMR values, patients were divided into 2 groups: those with AMR >2.7 mm Hg*s/cm and those with AMR ≤2.7 mm Hg*s/cm (Tables 1 and 2). Baseline patient characteristics between the 2 groups were similar in terms of age; sex; body mass index; history of hypertension, diabetes, and dyslipidemia; and smoking. No significant difference was observed in laboratory characteristics. The symptom-to-balloon time and the door-to-balloon time were 240.0 (IQR, 120.0–365.0) minutes and 54.0 (IQR, 48.0–60.0) minutes, respectively, and the differences between the 2 groups were not statistically significant (*P*=0.067 and *P*=0.139, respectively). The left anterior descending coronary artery was the most frequent infarct-related artery in both groups.

**Figure 2.**
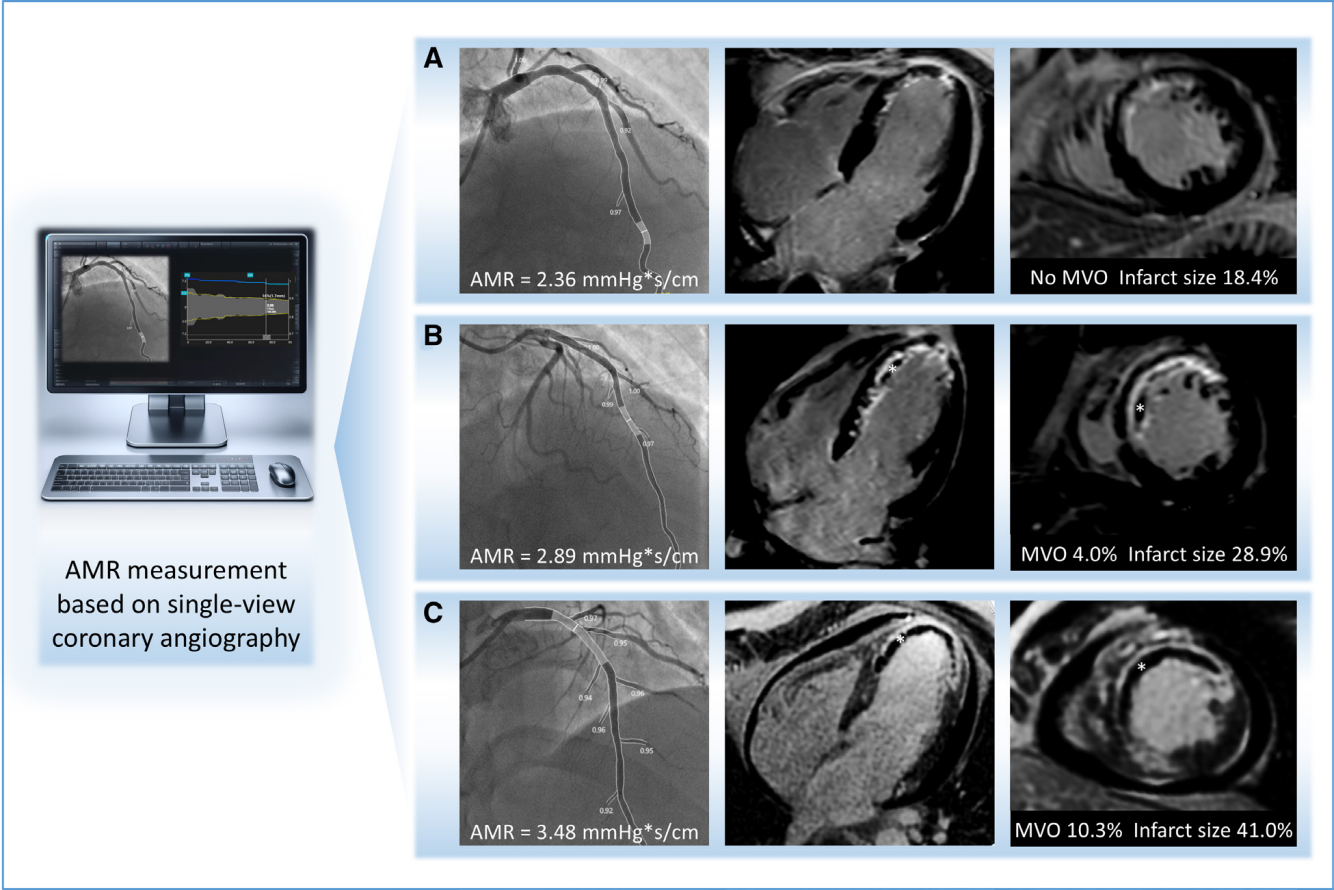
**Patients with ST-segment–elevation myocardial infarction with different angiography-derived microcirculatory resistance (AMR) values post-primary percutaneous coronary intervention (PPCI) of the infarct-related artery. A**, A patient (male, 51 years) with a preserved AMR of 2.36 mm Hg*s/cm, exhibiting no microvascular obstruction (MVO) and an infarct size of 18.4% on cardiac magnetic resonance; no heart failure (HF) observed during 61 months of follow-up. **B**, A patient (male, 48 years) with a moderately elevated AMR of 2.89 mm Hg*s/cm, displaying MVO at 4.0% and an infarct size of 28.9%; HF developed 65 days following PPCI. **C**, A patient (male, 50 years) with a significantly elevated AMR of 3.48 mm Hg*s/cm, showing significant MVO at 10.3% and a large infarct size of 41.0%; HF occurred 6 days following PPCI. * indicates the location of MVO.

Compared with patients with low AMR, those with high AMR had larger infarct size (30.3% [IQR, 22.5%–37.9] % versus 21.7 [IQR, 12.5%–28.6%]; *P*<0.001) and extent of MVO (3.4% [IQR, 1.5%–6.0%] versus 0% [IQR, 0%–1.0%]; *P*<0.001). Figure 2 illustrates case examples of patients with different AMR values after PPCI.

The Bland-Altman analysis revealed good agreement for AMR at both the intraobserver and interobserver levels, with a mean difference of 0.02 (95% CI, −0.20 to 0.24) mm Hg*s/cm for intraobserver measurements and 0.03 (95% CI, −0.24 to 0.30) mm Hg*s/cm for interobserver measurements (Figure 1E and 1F).

### Prognostic Implication of AMR in Patients With STEMI Post-PPCI

**Figure 3.**
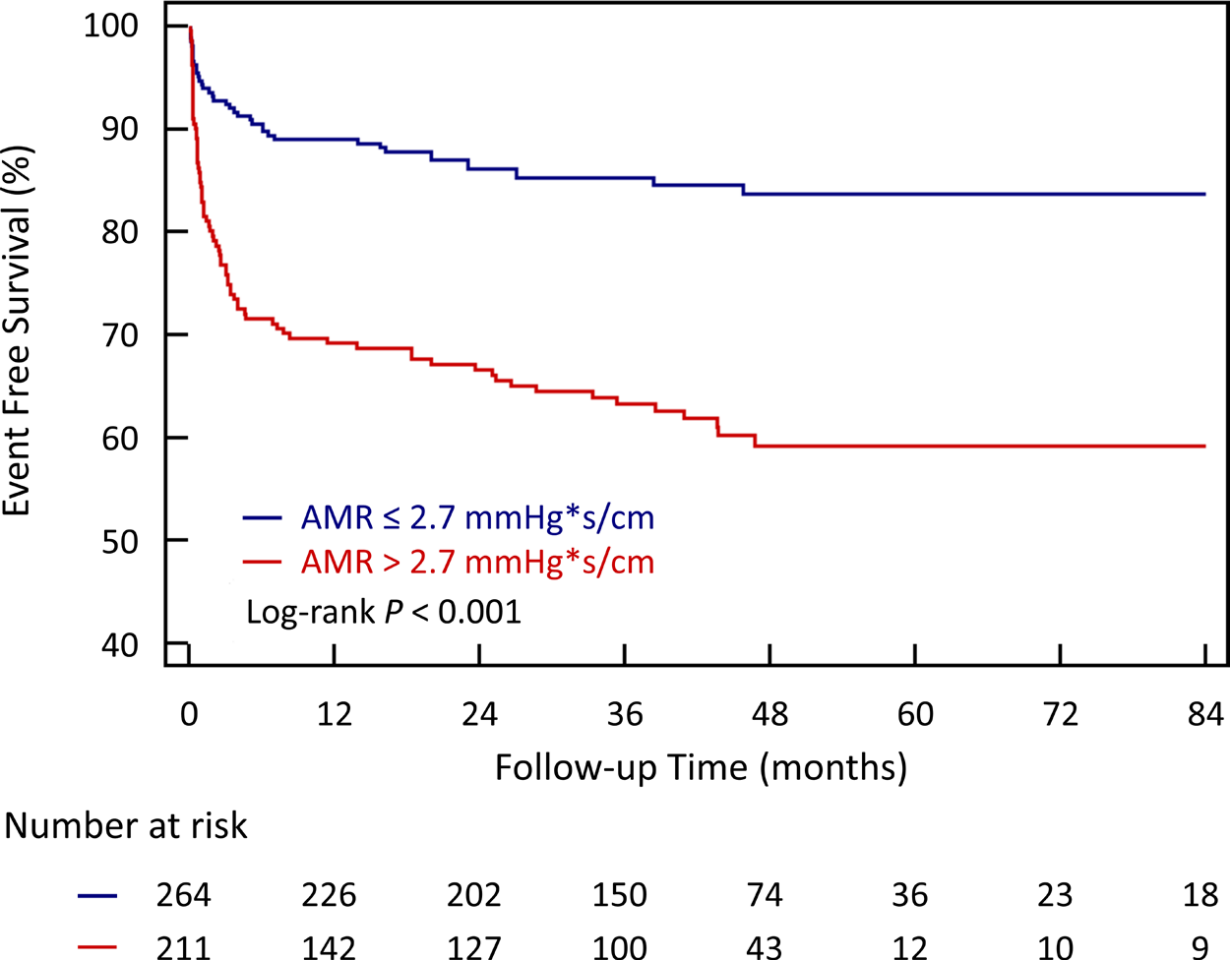
**Event-free survival in patients with ST-segment–elevation myocardial infarction following primary percutaneous coronary intervention according to angiography-derived microcirculatory resistance (AMR).** Patients with an elevated AMR >2.7 mm Hg*s/cm had a higher risk for heart failure than those with a preserved AMR value (log-rank *P*<0.001).

During a median follow-up of 37.3 (IQR, 13.8–48.0) months, new-onset HF occurred in 121 of 475 (25.5%) patients. Cardiac death occurred in 10 patients, all after HF onset. Patients with high AMR (>2.7 mm Hg*s/cm) had a higher probability of HF than those with low AMR (≤2.7 mm Hg*s/cm; log-rank *P*<0.001; Figure 3).

In the univariable Cox regression analyses, AMR, whether as a continuous (per 0.5-mm Hg*s/cm increase; hazard ratio, 1.44 [95% CI, 1.26–1.65]; *P*<0.001) or categorical (AMR >2.7 mm Hg*s/cm; hazard ratio, 2.90 [95% CI, 1.98–4.24]; *P*<0.001) variable, was associated with new-onset HF following PPCI (Table S2). After multivariable adjustment for age, symptom-to-balloon time, left anterior descending coronary artery as the infarct-related artery, LV ejection fraction, infarct size, and MVO extent, AMR remained independently associated with HF. For every 0.5-mm Hg*s/cm increase in AMR, the risk for HF increased by 29% (per 0.5-mm Hg*s/cm increase; adjusted hazard ratio, 1.29 [95% CI, 1.10–1.52]; *P*=0.002). Compared with patients with low AMR (≤2.7 mm Hg*s/cm), those with high AMR (>2.7 mm Hg*s/cm) had a 2.15-fold higher risk of HF (adjusted hazard ratio, 2.15 [95% CI, 1.43–3.22]; *P*<0.001; Table 3; Table S3).

**Table 3.**
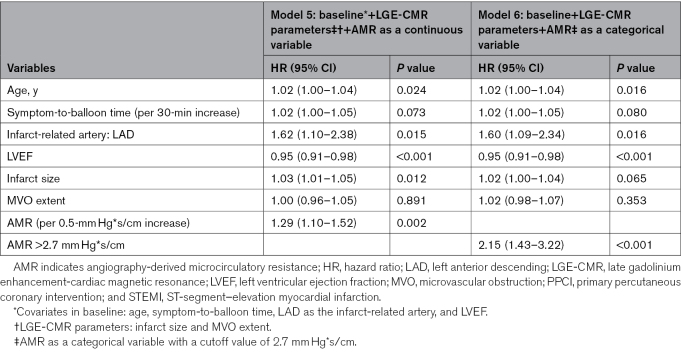
Multivariable Cox Regression Models for Predicting Heart Failure in Patients With STEMI Following PPCI (Models 5 and 6)

### Incremental Value of AMR for Predicting HF in Patients With STEMI Post-PPCI

Table 4 presents the prognostic performance of models for predicting HF in patients with STEMI post-PPCI. Model 1, which included significant traditional risk factors on univariable screening (age, symptom-to-balloon time, left anterior descending coronary artery as the infarct-related artery, and LV ejection fraction), demonstrated a C statistic of 0.703 (95% CI, 0.661–0.745). Adding AMR, either as a continuous or categorical variable, to model 1 significantly improved the C statistic. Specifically, adding AMR as a continuous variable (model 2) increased the C statistic from 0.703 to 0.735 (*P*=0.008) and resulted in a continuous NRI of 0.546 (*P*<0.001) and an IDI of 0.047 (*P*<0.001). When AMR was added as a categorical variable with a cutoff value of 2.7 mm Hg*s/cm (model 3), the C statistic increased from 0.703 to 0.740 (*P*=0.006), accompanied by a continuous NRI of 0.604 (*P*<0.001) and an IDI of 0.054 (*P*<0.001).

**Table 4.**
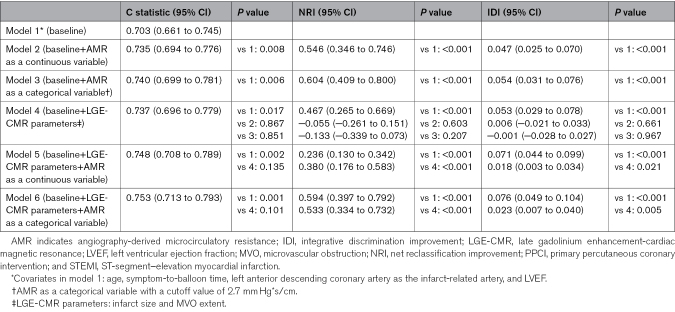
Prognostic Performance of Models for the Prediction of Heart Failure in Patients With STEMI Following PPCI

Adding LGE-CMR parameters to model 1 significantly improved the C statistic from 0.703 to 0.737 (*P*<0.001). No significant difference was observed in the prognostic performance of the model after adding LGE-CMR parameters compared with adding AMR, with similar C statistics (model 4 versus model 2: 0.737 versus 0.735; *P*=0.867; model 4 versus model 3: 0.737 versus 0.740; *P*=0.851). When AMR was added to the model with LGE-CMR parameters and traditional risk factors, the C statistic further increased and peaked (model 5: from 0.737 to 0.748; *P*=0.135; model 6: from 0.737 to 0.753; *P*=0.101) although these increases were not statistically significant. Nevertheless, the addition of AMR resulted in a continuous NRI of 0.380 (*P*<0.001) and an IDI of 0.018 (*P*=0.021) for model 5 and a continuous NRI of 0.533 (*P*<0.001) and an IDI of 0.023 (*P*=0.005) for model 6.

## Discussion

In this study, we investigated the capability of AMR for detecting MVO and its prognostic performance in predicting HF in patients with STEMI after successful PPCI. The main findings were that AMR exhibited good diagnostic performance in detecting MVO with an optimal cutoff value of 2.7 mm Hg*s/cm, and AMR was a powerful independent predictor of HF following PPCI and provided prognostic information incremental to traditional risk factors and LGE-CMR parameters.

### MVO in Patients With STEMI After Successful PCI

MVO is reported in over half of patients with STEMI despite thrombolysis in myocardial infarction flow grade of 3 following PCI.^6^ In the present study, MVO was observed in 253 of 475 patients (53.3%). Given the significance of periprocedural MVO as a potential therapeutic target in patients with STEMI for preventing ischemia/reperfusion injury and improving coronary microvascular function, a prompt on-site evaluation of MVO would be advantageous.^19^ CMR is the gold standard for assessing MVO.^7,8^ However, CMR cannot guide adjunctive therapies during revascularization to prevent persistent microvascular damage.^8,9^ IMR has been demonstrated as an available measure for assessing microvascular function immediately after stenting for STEMI and is associated with the extent of MVO.^9,10^ Nevertheless, the clinical adoption of IMR remains limited, particularly in patients with STEMI, due to the additional need for hyperemic agents and pressure-temperature sensor wires, which usually cause hypotension and prolong procedure time.^20^ Continuous thermodilution holds significant potential for assessing coronary microcirculation without requiring pharmacological vasodilation and with reduced operator bias.^21^ Post-PPCI microvascular resistance measured by this technique can predict coronary microvascular dysfunction in STEMI.^22^ However, it requires a guidewire, shares limitations with sensor wire-based indices, lacks reference values, and needs further validation.^20^

### AMR as a Wire- and Adenosine-Free Index for Assessing MVO

AMR, a novel angiography-derived index based on a single view, eliminates the need for a pressure wire and hyperemic agent.^15^ Fan et al^15^ demonstrated that AMR correlated well with invasive IMR, the standard reference, and exhibited good diagnostic accuracy. Our findings first revealed that AMR has good diagnostic performance in detecting MVO, with an optimal cutoff value of 2.7 mm Hg*s/cm, underscoring the potential of AMR for evaluating the coronary microvasculature status in patients with STEMI post-PPCI. This suggests that AMR is a promising index for identifying patients with MVO who may benefit from adjunctive therapies, such as small doses of intracoronary thrombolytics or vasodilators.^8,9^ In addition, AMR has demonstrated good intraobserver and interobserver reproducibilities,^15^ which aligns with the findings of our study. Notably, AMR overcomes the dependency on 2 angiographic views, which often fails to analyze approximately half of cases due to a lack of a second appropriate view.^16,17^ In our measurements, AMR was applicable in over 99.5% of cases. Moreover, our data revealed that the mean time required to measure AMR was only 2.26±0.90 minutes, highlighting its practicality in clinical settings, particularly during PPCI for STEMI. This could represent a significant advancement in precision medicine for patients with STEMI.

### Prognostic Implication of AMR for Predicting HF in Patients With STEMI Post-PPCI

HF following STEMI affects a significant proportion of patients, despite early rapid revascularization.^1,2^ In our study, HF developed in 121 of 475 patients (25.5%) during the follow-up period. Notably, an episode of new-onset HF in patients after STEMI is associated with a high mortality rate,^3,4^ emphasizing the importance of identifying patients at high risk of HF. This could enable prompt and aggressive optimization of therapy, thereby improving prognosis. One significant finding of the current study is that patients with higher AMR exhibited a significantly increased risk of HF. Particularly, AMR was found to be an independent predictor for new-onset HF following PPCI. For every 0.5-mm Hg*s/cm increase in AMR, HF risk increased by 29%. In addition, compared with patients with low AMR, those with elevated AMR >2.7 mm Hg*s/cm exhibited a 2.15-fold increased risk of HF, which may serve as a reference for risk stratification. This could facilitate an intuitive assessment of risk, helping clinicians efficiently evaluate prognosis and make informed therapeutic decisions. These findings confirm and extend the findings from previous reports, supporting the use of post-STEMI coronary microvascular dysfunction markers to stratify patients with STEMI at high risk after PPCI.^2,20^ Coronary microvascular dysfunction in STEMI is associated with suboptimal structural and functional myocardial recovery and repair, leading to adverse LV remodeling and an increased risk of HF.^5,11,13,23^ This provides a biologically plausible interpretation of the prognostic implications of AMR.

### Incremental Prognostic Value of AMR

We further revealed that AMR provided incremental prognostic value beyond traditional risk factors, which have been established as predictors previously.^24,25^ This supports integrating AMR into routine risk assessment for HF in patients with STEMI. In addition, adding AMR to traditional risk factors demonstrated prognostic performance similar to that of adding LGE-CMR parameters, reported to be associated with HF after STEMI.^5,6^ Moreover, AMR provided additional predictive value over both traditional risk factors and LGE-CMR parameters. Given that AMR does not require additional contrast injection and is accessible during PCI, AMR may be a more reasonable marker for refining early risk stratification in patients with STEMI.

Our study highlights that coronary physiology measured using AMR immediately post-PPCI can detect severe coronary microvascular dysfunction and provide important prognostic information.^2,20^ The favorable balance between ease of AMR use and diagnostic and prognostic performance emphasizes the importance of integrating this marker into routine clinical practice. Early identification of patients at high risk of HF will enable more aggressive therapeutic interventions and intensive follow-up to reduce HF incidence and improve long-term prognosis.^9^ Our study may inspire further research to evaluate strategies guided by AMR in reducing MVO and improving outcomes after PPCI for patients with STEMI.

### Limitations

Our study had some limitations. First, it was a retrospective single-center study and potentially subject to selection bias, limiting the generalizability of the findings; 2.7 mm Hg*s/cm proposed in our study serves as an initial exploratory reference for AMR in risk stratification. While valuable, further validation is necessary in more extensive and diverse populations before advocating for widespread clinical use. In addition, examining coronary-specific AMR cutoffs may be valuable. Second, we observed only a modest correlation between AMR and MVO/infarct size. This can be explained by the fact that MVO/IS on CMR represents an anatomic observation, while AMR is a functional measure of coronary microvascular status and viability, with differing assessment timings. Third, microcirculatory dysfunction in the territory of the infarct-related artery could be a dynamic condition. Consequently, a single measurement of AMR immediately after PPCI might not reflect the long-term microcirculatory status. Moreover, limiting assessment to AMR alone may miss a comprehensive evaluation of microvascular function. More comprehensive indices, such as coronary flow reserve, may play a role in risk-stratifying STEMIs and warrant further investigation.^26^ Finally, the lack of T2* data limited our ability to assess the correlation between AMR and intramyocardial hemorrhage, representing the most severe form of microvascular injury. Future studies should explore this correlation to improve understanding of microvascular injury following ischemia and reperfusion therapy.

### Conclusions

AMR showed good diagnostic performance in detecting MVO. Furthermore, AMR was a powerful independent predictor of HF in patients with STEMI post-PPCI, incremental to traditional risk factors and LGE-CMR parameters. Therefore, AMR shows promise as a predictor of HF in patients with STEMI following PPCI and may be used post-PPCI to inform therapeutic management and follow-up decisions.

## Article Information

### Sources of Funding

This work was supported by the National Natural Science Foundation of China (grants 82471929 and 82071875) and the Beijing Natural Science Foundation (grants 7212025 and 7222302).

### Disclosures

None.

### Supplemental Material

Supplemental Methods

Tables S1–S3

Figure S1

References 27–30
